# Pediatric Acute Myocarditis With Short-Term Outcomes and Factors for Extracorporeal Membrane Oxygenation: A Single-Center Retrospective Cohort Study in Vietnam

**DOI:** 10.3389/fcvm.2021.741260

**Published:** 2021-11-29

**Authors:** Ta Anh Tuan, Tran Dang Xoay, Phan Huu Phuc, Dau Viet Hung, Nguyen Trong Dung, Nguyen Ly Thinh Truong, Nguyen Van Thuan, Tran Minh Dien

**Affiliations:** ^1^Pediatric Intensive Care Unit, Vietnam National Children's Hospital, Hanoi, Vietnam; ^2^Children's Department, University of Medicine and Pharmacy, Vietnam National University, Hanoi, Vietnam; ^3^Department of Pediatric, Hanoi Medical University, Hanoi, Vietnam; ^4^Department of Cardiovascular Surgery, Children Heart Center, Vietnam National Children's Hospital, Hanoi, Vietnam; ^5^Surgical Intensive Care Unit, Vietnam National Children's Hospital, Hanoi, Vietnam

**Keywords:** acute myocarditis, cardiac arrhythmia, extracorporeal membrane oxygenation, mortality, pediatrics

## Abstract

**Objective:** Data on the management and outcomes of acute myocarditis treated with extracorporeal membrane oxygenation (ECMO) among low- and middle-income countries are limited. This study aimed to determine the short-term outcomes and also identify factors associated with ECMO use among children with acute myocarditis at a tertiary children's hospital in Vietnam.

**Methods:** A single-center, retrospective observational study was conducted between January 2016 and February 2021. Pediatric patients with acute myocarditis, aged 1 month to 16 years, were included.

**Results:** In total, 54 patients (male, 46%; median age, 7 years) with acute myocarditis were included; 37 of them received ECMO support. Thirty percent (16/54) of the patients died, and 12 of them received ECMO. Laboratory variables that differed between survivors and non-survivors included median left ventricular ejection fraction (LVEF) at 48 h (42 vs. 25%; *p* = 0.001), platelet count (304 g/L [interquartile range (IQR): 243–271] vs. 219 g/L [IQR: 167–297]; *p* = 0.014), and protein (60 g/dl [IQR: 54–69] vs. 55 [IQR: 50–58]; *p* = 0.025). Among patients who received ECMO, compared with the survivors, non-survivors had a low LVEF at 48 h (odds ratio (OR), 0.8; 95% confidence interval (CI): 0.6–0.9; *p* = 0.006) and high vasoactive-inotropic score (OR, 1.0; 95% CI: 1.0–1.0; *p* = 0.038) and lactate (OR, 2.8; 95% CI, 1.2–6.1; *p* = 0.013) at 24 h post-ECMO.

**Conclusions:** The case fatality rate among children with acute myocarditis was 30 and 32% among patients requiring ECMO support. Arrhythmia was an indicator for ECMO in patients with cardiogenic shock.

## Introduction

Acute myocarditis in children is a rare but life-threatening disease that can cause sudden death (1). Early diagnosis and prompt intervention play a pivotal role in the management of the disease. Current treatment is mainly supportive (2). Acute myocarditis may progress very quickly and eventually lead to cardiogenic shock requiring resuscitation and administration of intravenous inotropic agents (1, 2). Extracorporeal membrane oxygenation (ECMO) therapy is indicated for patients who are non-responsive to medical therapy and have an increased serum lactate concentration and evidence of organ failure (3). ECMO is an effective treatment modality in managing critical cases, with reported success rates of 50–83% (4, 5). Factors associated with mortality include arrhythmia (3), dialysis (6), and end-organ hypoperfusion.

There are numerous studies on the deployment of ECMO for acute myocarditis in children in developed countries. Data on the management and outcomes of acute myocarditis treated with extracorporeal membrane oxygenation (ECMO) among low- and middle-income countries are limited.

This study aimed to determine short-term outcomes and identify factors associated with ECMO use among children with acute myocarditis at a tertiary children's hospital in Vietnam.

## Materials and Methods

### Patients and Case Definitions

A single-center, retrospective observational study was conducted between January 2016 and February 2021 in the pediatric intensive care unit (PICU) of Vietnam National Children's Hospital. Informed consent was obtained from parents/guardians. The study was approved by the Ethics Council of Vietnam National Children's Hospital (approval number: 331-BVNTW-VNVSKTE).

**Figure 1:**
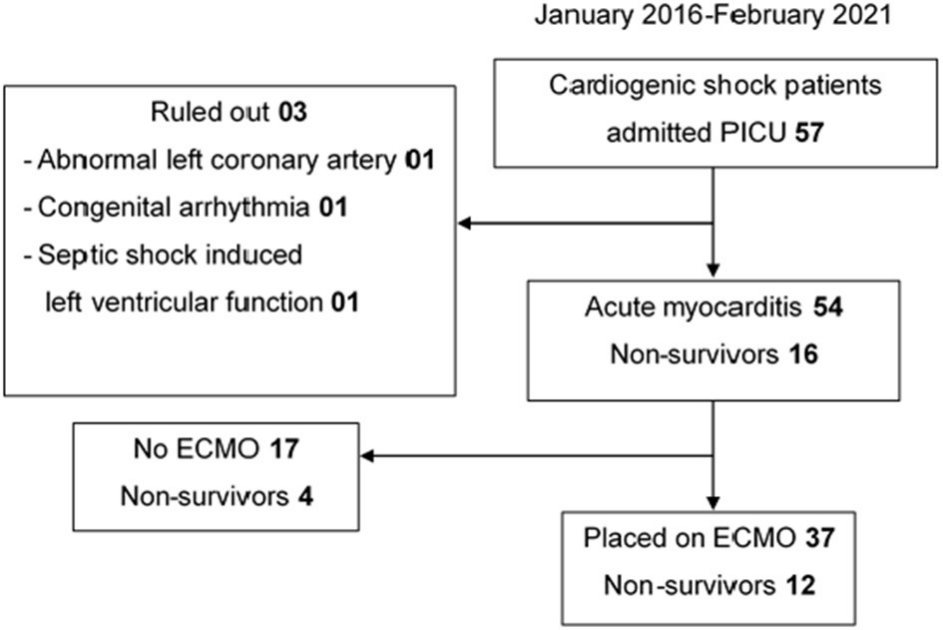
Flowchart of enrolled cases. ECMO, extracorporeal membrane oxygenation.

Consecutive pediatric patients aged 1 month to 16 years who met the criteria for acute myocarditis were included (Figure 1). The definition and diagnostic criteria for acute myocarditis were adapted from Sagar et al. (7). Acute myocarditis was diagnosed for a patient who had clinical features of possible myocardial injury with cardiovascular symptoms and at least one of the following findings: (i) elevated biomarkers of cardiac injury, (ii) electrocardiogram (ECG) findings suggestive of cardiac injury, and (iii) abnormal cardiac function on echocardiogram in the absence of endomyocardial biopsy. Notably, magnetic resonance imaging (MRI) was not widely used to evaluate the cardiac function at our center.

Demographic, clinical, and laboratory data were collected as part of routine care. Demographic and clinical variables included age, sex, weight, presence of cough, runny nose, anorexia, abdominal pain, vomiting, chest pain, syncope, seizure, palpitation, fever, pallor, hypotension, gallop rhythm, heart murmur, edema, hepatomegaly, pulmonary edema, and time from the onset of symptoms to PICU admission. Laboratory variables included creatine kinase-MB (CK-MB), troponin I, N-terminal pro B type natriuretic peptide (NT-proBNP), the total white blood cell (WBC) neutrophil, lymphocyte and platelet counts (PLT), C-reactive protein (CRP), serum urea, creatinine, alanine aminotransferase (ALT), aspartate aminotransferase (AST), albumin, and protein levels. Cardiac data obtained before and after ECMO included left ventricular ejection fraction (LVEF), left ventricular dilation, left ventricular diastolic diameter, abnormal segmental wall motion, pericardial effusion, pleural effusion, abdominal effusion, and ECG findings. Real-time polymerase chain reaction (RT-PCR) was used to evaluate the presence of viral antigens in serum and respiratory tract aspirates. Adjunct therapies included defibrillation, pacemaker, hemodialysis, intravenous immunoglobulin (IVIG), and amiodarone. We did not use immunosuppressive medications for patients in this cohort.

### Extracorporeal Membrane Oxygenation

Indications for ECMO therapy were according to the Extracorporeal Life Support Organization (8). The patients who qualified for ECMO had at least one of the following: (i) an oxygen index >40 for a total duration of 4 h, (ii) cardiogenic shock with arrhythmia requiring supportive inotropes, (iii) cardiogenic shock without arrhythmia requiring supportive inotropes (vasoactive-inotropic score [VIS] >40), and (iv) lactic acidosis that did not improve with vasoactive-inotropic agents and pharmacologic therapies. For patients with a bodyweight of ≤ 20 kg, we used the Maquet machine with the Rotaflow pump module (Maquet Cardiopulmonary, Rastatt, Germany) and an oxygenator; a Maquet PLS-i oxygenator (Maquet Cardiopulmonary, Rastatt, Germany) was only used for patients with a bodyweight of ≥20 kg. Bio-Medicus cannulas (Medtronic, MN, USA) were used, and cannula size was selected according to the patient's body weight. We collected the following variables regarding ECMO therapy: ECMO mode, ECMO duration, time (in hours) from PICU admission to ECMO initiation, cannula sites (neck or groin), and the flow at 4 and 24 h on ECMO. Prior to and during ECMO therapy, clinical and laboratory variables, including heart rate and blood pressure, cardiac arrest, VIS, arterial blood gas and mixed venous oxygen saturation (ScvO_2_), and blood lactate level, were collected. After the initiation of ECMO, hemorrhage, cannula deviation, clots in the oxygenator, hemolysis, and infection were considered complications of ECMO.

Criteria for the termination of ECMO were as follows: (i) recovery from shock, (ii) stable hemodynamics and overall condition upon decrease of ECMO to 30% of its maximum flow, (iii) normal chest X-ray, (iv) pH within the range of 7.35–7.45, and (v) echocardiogram-confirmed recovery of ventricular function. We initially decreased the blood flow of the ECMO support to zero for 5 min and discontinued afterwards if the patient remained stable.

The VIS was calculated as follows: dopamine dose (μg/kg/min) + dobutamine dose (μg/kg/min) + 100 × epinephrine dose (μg/kg/min) + 10 × milrinone dose (μg/kg/min) + 10,000 × vasopressin dose (unit/kg/min) + 100 × norepinephrine dose (μg/kg/min) (9).

Short-term outcomes, including survival and death from all causes, were assessed at discharge from the PICU.

### Statistical Analysis

Continuous variables are expressed as the median and interquartile range (IQR), while categorical variables are expressed as a percentage. Comparisons between groups were evaluated using logistic regression analysis. Non-survival, a secondary outcome, is expressed as the odds ratio (OR) with corresponding 95% confidence intervals (CIs). Multivariable logistic regression analysis was not performed due to the small sample size. The significance level was set at a two-sided *p* < 0.05. IBM SPSS Statistics Software Version 20 (IBM Corp., Armonk, NY, USA) was used for statistical analyses.

## Results

### Patients

In total, 54 patients (male, 46%), median age of 7 years (IQR, 3–10), with acute myocarditis were included. The median weight was 18.5 kg (IQR, 10–29). The median length of PICU stay was 14 days (IQR, 12–19; range, 2–61 days).

### Signs and Symptoms

The median time from the onset of symptoms to PICU admission was 3 days (IQR, 2–4). Signs and symptoms included cough (35%), runny nose (18%), anorexia (53%), abdominal pain (33%), vomiting (61%), chest pain (32%), syncope (11%), seizure (9%), palpitation (21%), and fever (60%). Signs of myocarditis on the day of PICU admission were pallor (67%), hypotension (91%), gallop rhythm (42%), heart murmur (4%), edema (4%), hepatomegaly (70%), and pulmonary edema (7%). The median pediatric risk of mortality score was 9 (Table 1).

**Table 1:** Comparison between survivors and non-survivors in terms of demographics, symptoms, and signs at the pediatric intensive care unit admission.

**Variables**	**Total** **(*n* = 54)**	**Survivors** **(*n* = 38)**	**Non-survivors** **(*n* = 16)**	**OR** **(95% CI)**	***p* ^**a**^**
Age (yr)	7 (3–10)	7 (3–9)	4 (1–10)	0.9 (0.8–1.1)	0.245
Male	25 (44%)	18 (47%)	7 (44%)	0.9 (0.3–2.8)	0.808
Weight at admission (kg)	18.5 (10–29)	20.5 (12.8–29.3)	13.5 (6.6–27.8)	1 (0.9–1)	0.324
Days from onset to PICU admission	3 (2–4)	3 (2–4)	3 (2–4.8)	1.1 (0.8–1.6)	0.442
Cough	20 (35%)	13 (34%)	7 (44%)	1.5 (0.5–4.9)	0.509
Runny nose	10 (18%)	5 (13%)	5 (31%)	3 (0.7–12.3)	0.128
Anorexia	30 (53%)	20 (53%)	10 (63%)	1.5 (0.5–5)	0.506
Abdominal pain	19 (33%)	14 (37%)	5 (31%)	0.8 (0.2–2.7)	0.695
Vomiting	35 (61%)	25 (66%)	10 (63%)	0.9 (0.3–2.9)	0.817
Chest pain	18 (32%)	14 (37%)	4 (25%)	0.6 (0.2–2.1)	0.402
Syncope	6 (11%)	4 (11%)	2 (13%)	1.2 (0.2–7.4)	0.833
Seizure	5 (9%)	4 (11%)	1 (6%)	0.6 (0.1–5.5)	0.624
Palpitation	12 (21%)	9 (24%)	3 (19%)	0.7 (0.2–3.2)	0.691
Fever (>37.5°C)	34 (60%)	23 (61%)	11 (69%)	1.4 (0.4–5)	0.569
Pallor	38 (67%)	26 (68%)	12 (75%)	1.4 (0.4–5.2)	0.630
Hypotension	52 (91%)	36 (95%)	16 (100%)	–	0.999
Gallop rhythm	24 (42%)	18 (47%)	6 (38%)	0.7 (0.2–2.2)	0.506
Heart murmur	2 (4%)	1 (3%)	1 (6%)	2.5 (0.1–42.1)	0.533
Edema	2 (4%)	1 (3%)	1 (6%)	2.5 (0.1–42.1)	0.533
Hepatomegaly	40 (70%)	29 (76%)	11 (69%)	0.7 (0.2–2.5)	0.564
Pulmonary edema	4 (7%)	2 (5%)	2 (13%)	2.6 (0.3–20.1)	0.368
Pericardial effusion	5 (9%)	5 (13%)	0 (0%)	–	0.999
Plural effusion	14 (25%)	8 (21%)	5 (31%)	0.8 (0.2–2.5)	0.675
Abdominal effusion	6 (11%)	3 (8%)	3 (19%)	0.7 (0.2–3.2)	0.691
Pediatric Risk of Mortality Score 2	9 (8–12)	9 (7–11)	11 (8–16)	1.1 (1.0–1.3)	0.122
Arrhythmia	37 (65%)	27 (71%)	10 (63%)	0.7 (0.2–2.3)	0.538
ECMO used	37 (65%)	25 (66%)	12 (75%)	1.6 (0.4–5.8)	0.508

*ECMO, Extracorporeal Membrane Oxygenation; OR, odds ratio, CI, confidence interval*.

a *p < 0.05 was considered significant. The data are presented as the number (%) or median (interquartile). Comparison between groups was analyzed by logistic regression analysis and expressed as OR with corresponding 95% CI*.

### Laboratory Data and Clinical Course

Laboratory variables collected within 24 h of PICU admission, organ dysfunction, and therapies are shown in Table 2. Laboratory variables that differed significantly between survivors and non-survivors included LVEF at 48 h (42 vs. 25%; *p* = 0.001). PLT count and serum protein levels of non-survivors were significantly lower than that of survivors (*p* = 0.014 and 0.025, respectively). There were no significant differences between survivors and non-survivors for other laboratory variables.

**Table 2:** Comparison between survivors and non-survivors in terms of laboratory data at admission, organ dysfunction, and treatments during the pediatric intensive care unit stay.

**Variables**	**Total** **(*n* = 54)**	**Survivors** **(*n* = 38)**	**Non-survivors** **(*n* = 16)**	**OR** **(95% CI)**	***p* ^**a**^**
Creatin kinase-MB (U/L)	91 (44–173)	91 (45–150)	78 (26–251)	1.0 (1.0–1.0)	0.834
Troponin I (ng/mL)	20.7 (3.8–132.7)	20.7 (3.8–96.7)	22.2 (1.4–298.3)	1.0 (1.0–1.0)	0.271
N-terminal pro B type natriuretic peptide (pmol/L)	3,090 (1,625–5,930)	3,090 (1,286–4,934)	3,593 (1,759–7,950)	1.0 (1.0–1.0)	0.360
White blood cell (× 10^9^/L)	14 (10.6–20)	13.6 (10.3–19.6)	14.7 (11.6–20.7)	1.0 (0.9–1.1)	0.204
Neutrophil (× 10^9^/L)	8.7 (5.7–16)	8.7 (5.7–14.2)	8.6 (5.8–17.6)	1.0 (0.9–1.1)	0.232
Lymphocyte (× 10^9^/L)	2.7 (1.8–5)	2.7 (1.7–4.7)	3.5 (2.2–5.5)	1.1 (0.9–1.2)	0.202
Platelet (× 10^9^/L)	280 (214–356)	304 (243–371)	219 (167–297)	**0.9 (0.9–0.9)**	***0.014***
C-reactive protein (mg/L)	8.8 (1.8–22.4)	10.8 (1.8–24.4)	7.9 (1.8–9.9)	1.0 (0.9–1.0)	0.544
Urea (mmol/L)	7.9 (6.4–10.2)	8 (6–12)	8 (7–10)	0.9 (0.8–1.0)	0.528
Creatinine (μmol/L)	69 (56–81)	68 (55–78)	76 (63–83)	1.0 (1.0–1.0)	0.599
Alanine aminotransferase (IU/L)	61 (27–162)	43 (25–96)	119 (60–543)	1.0 (1.0–1.0)	0.326
Aspartate transaminase (IU/L)	172 (99–469)	141 (81–457)	376 (134–1134)	1.0 (1.0–1.0)	0.259
Albumin (g/L)	36 (33–39)	36 (34–39)	35 (32–39)	0.9 (0.8–1.1)	0.330
Protein (g/L)	57 (53–67)	60 (54–69)	55 (50–58)	**0.9 (0.8–1.0)**	***0.025***
Left ventricular ejection fraction (%)	35 (26–42)	40 (30–46)	31 (24–38)	1.0 (0.9–1.0)	0.148
Left ventricular diastolic diameter (mm)	40 (36–44)	40 (36.5–43)	39.8 (35.8–49.5)	1.0 (1.0–1.1)	0.307
Left ventricular ejection fraction post 48 h (%)	37 (29–46)	42 (31.5–49.5)	25 (20–30)	**0.8 (0.7–0.9)**	***0.001***
Troponin I post 48 h (ng/mL)	2.7 (0.7–11.5)	2.3 (0.7–7.5)	6.6 (0.4–36.9)	1.0 (1.0–1.0)	0.690
N-terminal pro B type natriuretic peptide post 48 h (pmol/L)	2,776 (1,650–3,886)	2,776 (13.4–3,832)	2,584 (1,701–8,011)	1.0 (1.0–1.0)	0.121
Creatinine highest (μmol/L)	77 (64–115)	74 (59–95)	86 (69–160)	1.0 (1.0–1.0)	0.883
Alanine aminotransferase highest (IU/L)	211 (84–842)	124 (67–574)	436 (150–1625)	1.0 (1.0–1.0)	0.302
Aspartate transaminase highest (IU/L)	508 (143–1,715)	271 (114–1,046)	1,275 (591–4,476)	1.0 (1.0–1.0)	0.082
Defibrillation	17 (30%)	10 (26%)	7 (44%)	2.2 (0.6–7.4)	0.212
Pacemaker during the PICU stay	10 (18%)	10 (26%)	0 (0%)	-	0.999
Continuous renal replacement therapy	20 (35%)	12 (32%)	8 (50%)	2.2 (0.7–7.2)	0.205
Intravenous Immunoglobulin with vasoactive-inotropic score at admission	13 (23%) 20 (13.7–35)	8 (21%) 17.5 (10.6–31.2)	5 (31%) 27.5 (21.2–87.5)	1.7 (0.5–6.3) 1.1 (0.9–1.2)	0.426 0.332
Amiodarone	13 (23%)	10 (26%)	3 (19%)	0.6 (0.2–2.8)	0.554
Mechanical ventilation days	10 (7–13)	10 (7–12)	10 (4–16)	1.0 (0.9–1.1)	0.949
PICU stay (d)	14 (12–19)	14 (13–20)	11 (4–19)	0.9 (0.9–1.0)	0.134

*OR, odds ratio*.

a *p < 0.05 was considered significant. PICU, pediatric intensive care unit. The data are presented as the number (%) or median (interquartile range). Comparison between groups was analyzed by logistic regression analysis and expressed as OR with corresponding 95% CI. The p-values were less than 0.05*.

Overall, 37 patients presented with ECG abnormalities on admission to the PICU. There were six patients with complete atrioventricular (AV) block, two with first degree AV block, one with second degree AV block, one with ventricular fibrillation, 15 with ventricular tachycardia, two with ST-elevation, two with right branch block, two with low voltage QRS, and six with premature ventricular contractions.

### Etiology of Acute Myocarditis

Nine patients had positive microbiological diagnoses; five were infected with enteroviruses, one with combined enterovirus and adenovirus, one with adenovirus, one with bocavirus, and one with *Staphylococcus aureus*.

### Comparison Between Patients With and Without Extracorporeal Membrane Oxygenation

In total, 37 patients received veno-arterial-ECMO therapy. The mortality rate of cases was 32% (12/37). Table 3 shows the characteristics between the ECMO group and the non-ECMO group. The presence of arrhythmia was significantly higher among patients receiving ECMO therapy (OR, 26.8; 95% CI: 5.8–123.5; *p* < 0.001). There were differences between the two groups for age and body weight (*p* = 0.019 and 0.025, respectively).

**Table 3:** Comparison of factors between patients with and without extracorporeal membrane oxygenation.

**Variables**	**ECMO** **(*n* = 37)**	**Non-ECMO** **(*n* = 17)**	**OR** **(95% CI)**	***p* ^***a***^**
Age (yr)	7 (5.5–10)	2 (1–9)	**1.2 (1.0–1.4)**	***0.019***
Male	19 (51%)	6 (35%)	1.9 (0.6–6.3)	0.275
Dead	12 (32%)	4 (24%)	1.6 (0.4–5.8)	0.508
Weight on admission PICU (kg)	22 (15.5–30)	10 (7–24.8)	**1.1 (1.0–1.1)**	***0.025***
Days from onset to PICU admission	3 (2–4)	3 (2–4)	1.1 (0.8–1.5)	0.773
Vasoactive-Inotropic Score at 24 h	35 (20–120)	20 (15–45)	1.0 (1.0–1.0)	0.053
Arrhythmia	33 (89%)	4 (24%)	**26.8 (5.8–123.5)**	***<0.001***
Creatin kinase-MB (U/L)	104 (72–233)	65 (34–111)	1.0 (1.0–1.0)	0.081
Troponin I (ng/mL)	49.1 (6.4–412.9)	4.9 (1.2–8.3)	1.0 (1.0–1.0)	0.143
N-terminal pro B type natriuretic peptide (pmol/mL)	2,624 (1,290–4,138)	4,771 (3,078–7,397)	1.0 (1.0–1.0)	0.289
Left ventricular ejection fraction (%)	33 (25.5–43)	39 (30.5–42.5)	1.0 (0.9–1.0)	0.748
Creatinine (μmol/L)	70 (62–83)	56 (43–79)	1.0 (1.0–1.1)	0.089
Alanine aminotransferase (IU/L)	78 (31–490)	48 (21–100)	1.0 (1.0–1.0)	0.230
Aspartate transaminase (IU/L)	285 (109–1,081)	138 (66–227)	1.0 (1.0–1.0)	0.118
Continuous Renal Replacement Therapy	13 (35%)	7 (41%)	0.8 (0.2–2.5)	0.670

*OR, odds ratio*.

a *p < 0.05 was considered significant. The data are presented as the number (%) or median (interquartile range). Comparison between groups was analyzed by logistic regression analysis and expressed as OR with corresponding 95% CI. The p-values were less than 0.05*.

The severity and hemodynamic characteristics of children receiving ECMO therapy are shown in Table 4. The variables that differed between non-survivors and survivors were LVEF at 48 h (OR, 0.8; 95% CI: 0.6–0.9; *p* = 0.006), VIS at 24 h post- ECMO therapy (OR, 1.0; 95% CI: 1.0–1.0; *p* = 0.038), and lactate level at 48 h post-ECMO therapy (OR, 2.8; 95% CI: 1.2–6.1; *p* = 0.013).

**Table 4:** Comparison between survivors and non-survivors in terms of laboratory values before and 24 h after extracorporeal membrane oxygenation.

**ECMO parameters**	**Total** **(*n* = 37)**	**Survivors** **(*n* = 25)**	**Non-survivors** **(*n* = 12)**	**OR** **(95% CI)**	***p***
Cardiac arrest before ECMO	10 (27%)	5 (20%)	5 (42%)	2.9 (0.6–12.9)	0.173
Time from admission to ECMO (hr)	6 (3–12)	6 (3.5–14.5)	5 (3–11)	1.0 (0.9–1.0)	0.508
ECMO duration (d)	7 (5–11.5)	7 (6–9.5)	10 (3–17)	1.1 (1.0–1.2)	0.208
Cannula site (neck)	30 (81%)	21 (84%)	9 (75%)	0.6 (0.1–3.1)	0.516
Cannula site (groin)	7 (19%)	4 (16%)	3 (25%)	1.8 (0.3–9.5)	0.516
Flow at 4 h (ml/kg/min)	87 (69–101)	87 (71–100)	88 (62–122)	1.0 (1.0–1.0)	0.250
Flow at 24 h (ml/kg/min)	88 (70–106)	79 (70–98)	96 (74–122)	1.0 (1.0–1.0)	0.243
Heart rate pre-ECMO (beats/min)	150 (133–167)	150 (121–165)	153 (141–184)	1.0 (1.0–1.0)	0.546
Systolic blood pressure pre-ECMO (mmHg)	92 (74–100)	92 (74–108)	88 (72–97)	1.0 (0.9–1.0)	0.187
Diastolic blood pressure pre-ECMO (mmHg)	51 (42–60)	51 (42–60)	49 (34–65)	1.0 (1.0–1.0)	0.687
Vaso-Inotropic Score pre-ECMO	55 (35–121.3)	47.5 (35–113.8)	70 (20–137.5)	1.0 (1.0–1.0)	0.493
Left ventricular ejection fraction (%)	33 (25.5–43)	40 (28–49)	30 (23.5–35.9)	1.0 (0.9–1.0)	0.246
Left ventricular ejection fraction post 48 h (%)	38 (29–46)	44 (35–48.4)	25 (20–30)	**0.8 (0.6–0.9)**	***0.006***
pH pre-ECMO	7.35 (7.26–7.41)	7.35 (7.29–7.41)	7.35 (7.18–7.42)	0.1 (0.0–22)	0.331
${\text{HCO}}_{3}^{-}$ pre-ECMO (mmol/L)	18.2 (13.9–22.2)	17.6 (14.2–21.6)	19 (12.7–24.3)	1.0 (0.9–1.2)	0.615
Lactate pre-ECMO (mmol/L)	3.6 (2–5.8)	3.6 (1.8–6.5)	3.8 (2.3–5.5)	1.0 (0.8–1.2)	0.922
Alanine aminotransferase pre-ECMO (IU/L)	78 (31–388)	49 (29–146)	149 (72–603)	1.0 (1.0–1.0)	0.949
Aspartate transaminase pre-ECMO (IU/L)	303 (103–997)	149 (97–480)	523 (163–1200)	1.0 (1.0–1.0)	0.909
Mixed venous oxygen saturation (SvO_2_) pre-ECMO (%)	58 (45–66.5)	58 (44.5–66.5)	60 (47–66.8)	1.0 (1.0–1.1)	0.615
Mixed venous oxygen saturation (SvO_2_) post-ECMO 24 h (%)	67 (65–69.5)	67 (65–70)	65 (63–67.8)	0.8 (0.6–1.0)	0.078
Oxygen index pre-ECMO	3 (2–5)	3 (2.5–5)	3.5 (2–5)	0.9 (0.7–1.2)	0.525
Vaso-inotropic Score post-ECMO 24 h	30 (12.8–118.8)	15 (10–63.8)	95 (28.1–147.5)	**1.0 (1.0–1.0)**	***0.038***
pH post-ECMO	7.35 (7.30–7.40)	7.36 (7.31–7.40	7.35 (7.27–7.43)	0.2 (0.0–229.6)	0.656
${\text{HCO}}_{3}^{-}$ post-ECMO (mmol/L)	23.2 (20.1–27.1)	23 (20.1–26.9)	23.4 (19.8–27.2)	1.0 (0.8–1.1)	0.819
Lactate post-ECMO 48 h (mmol/L)	1.3 (0.8–2.3)	1.1 (0.7–1.5)	2.1 (1.3–4.1)	***2.8 (1.2–6.1)***	***0.013***

*OR, odds ratio. The data are presented as the number (%) or median (interquartile range). Comparison between groups was analyzed by logistic regression analysis and expressed as OR with corresponding 95% CI. Statistical significance is set at p < 0.05. The p-values were less than 0.05*.

Nine (24%) patients experienced complications associated with ECMO therapy. Three patients had oxygenator clotting and required the oxygenator to be exchanged. One patient developed a swollen leg associated with the groin cannula. One patient experienced a hemorrhage at the cannula site, while another developed a pleural hemorrhage that caused the compression of the left ventricle. A patient with ventricular thrombosis, created by a swirling current in the heart chambers, died after decannulation due to failure of cardiac function. Two patients exhibited brain death.

### Adjunct Therapies

Adjunct therapies included mechanical ventilation, inotropes, continuous venovenous hemofiltration, sedation, defibrillation, pacemaker, IVIG, cordarone, and ECMO. All the patients required invasive mechanical ventilation support and the administration of inotropes and vasopressors, such as dopamine, dobutamine, epinephrine, norepinephrine, and milrinone.

### Overall Outcomes

Sixteen patients (30%) died after discharge from the PICU. Among patients requiring ECMO, 25 patients survived (68%). The median length of PICU stay for survivors in the ECMO and non-ECMO groups was 15 (IQR, 13–20) and 13 (IQR, 8–15) days, respectively.

Six patients (16%) had a systemic infection during their ECMO treatment and PICU stay. Cultured samples were obtained from the blood and endotracheal tube. The organisms found were *Acinetobacter baumannii* in four patients, *Pseudomonas aeruginosa* in one, and *Moraxella catarrhalis* in one.

## Discussion

This study described ECMO application and short-term outcomes for 54 children with acute myocarditis in a single center from a low- and middle-income country. Overall, the case fatality rate was 30%. Among patients receiving ECMO, the case fatality rate was 32%, which was similar to the findings from previous studies conducted in high-income countries and Asia (3, 5, 10, 11). To the best of our knowledge, our case series is the first study with the highest case number so far conducted in a low- and middle-income country.

Similar to previous reports, gastrointestinal symptoms, such as anorexia, abdominal pain, and vomiting, were common (12, 13). All patients had cardiac-specific signs, such as pallor and hypotension, upon admission to the PICU. Other cardiac-specific signs included gallop rhythm and hepatomegaly, which occurred in the majority of cases. Overall, four patients had pulmonary edema and hepatomegaly at admission to PICU; of these, one had cardiac arrest, and two demised. Fluid resuscitation, with 5 ml/kg, was conservatively administered if needed; additionally, we promptly administered vasoactive-inotropic agents and established ECMO in the patients. After they were placed on ECMO, the symptom of pulmonary edema resolved. When pulmonary edema was persistent during ECMO, left ventricular compression was resolved using inotropic agents, including dopamine, dobutamine, and milrinone to increase contractility. If aortic valve opening and clinical improvement was not seen, an atrial septal defect creation should be considered (14, 15). We did not histologically confirm acute myocarditis in the children because of the lack of endocardial biopsy in the PICU. Hence, the diagnosis of acute myocarditis was based on the diagnostic criteria of Sagar et al. (7). Other differential diagnoses considered were heart failure secondary to primary arrhythmia, and anomalous left coronary artery from the pulmonary artery.

Similar to previous studies (1, 16, 17), our results show that non-survivors had significantly lower LVEF on admission to the PICU than survivors and did not recover despite receiving ECMO support. Other abnormalities on echocardiography, also described in a previous study (18), included segmental wall motion and pericardial effusion. An echocardiogram is a commonly used valuable tool for evaluating left ventricular structure and function. In addition, ultrasound detection could also aid in the diagnosis of pleural and abdominal effusion.

Most patients in this study had arrhythmias that caused cardiac failure and resulted in an increased risk of sudden death, which was why ECMO was indicated. Rajagopal et al. (4) found that the presence of cardiac arrhythmias requiring therapy while on ECMO support was associated with an ~3-fold increase in mortality.

In this study, non-survivors had greater odds of VIS and serum lactate level at 24 h post-ECMO, which were signs of progressive organ failure. Together with irreversible cardiac dysfunction, they lead to multi-organ failure, this was the main cause of death in this study population. In another study, Duncan et al. (3) showed that elevated markers of end-organ function, such as creatinine, liver enzymes, and serum lactate, prior to and immediately after ECMO support were predictive of increased mortality. According to near-infrared spectroscopy, which obtained a continuous measurement of cerebral saturation (19), two patients experienced brain death.

Due to the inconclusive evidence of IVIG efficacy, we did not routinely administer IVIG for patients with myocarditis (20, 21). We only used IVIG according to the protocol in two cases with enterovirus infections, whereas empirical treatment was adopted for the remaining cases with high dose 2 g/kg/ 24 h. There was no statistically significant difference between the survivors and non-survivors who were treated with IVIG. We did not use immunosuppressive medications such as corticoids for patients in this cohort. The strongest evidence for immunosuppressive therapy occurs in cardiac sarcoidosis, giant-cell myocarditis, and autoimmune rheumatic disease (22–24). However, recent studies in adults and pediatrics reported that immunosuppressive therapy with IVIG and/or high dose methylprednisolone was associated with rapid LVEF recovery, short duration of ECMO support, and low mortality rate in fulminant myocarditis (25, 26). Nevertheless, further well-designed studies are required to investigate this issue in the pediatric population.

The primary limitation of this study was the retrospective, single-center cohort and the small sample size. Consequently, the differences between the groups were not statistically significant. Another limitation of this study was the lack of follow-up on dilated cardiomyopathy.

## Conclusion

Overall, the case fatality rate of children with acute myocarditis at a single PICU was 30 and 32% among patients requiring ECMO therapy. Non-survivors had lower odds of LVEF at 48 h and lower PLT count and serum protein levels than survivors. Arrhythmia was an indication for ECMO therapy.

## Data Availability Statement

The raw data supporting the conclusions of this article will be made available by the authors, without undue reservation.

## Ethics Statement

The studies involving human participants were reviewed and approved by the Ethics Council of Vietnam National Children's Hospital (approval number: 331-BVNTW-VNVSKTE). Written informed consent to participate in this study was provided by the participants' legal guardian/next of kin.

## Author Contributions

TT, TX, PP, DH, and TD contributed to the conception and design of the study. TT, TX, and NVT collected the clinical information. TX wrote the first draft of the manuscript. TT, TX, and PP wrote sections of the manuscript. All authors have revised, read, and approved the final version of the manuscript.

## Funding

This work was supported, in part, by Grant-in-Aid from the Vietnam National University—Hanoi, Vietnam (QGSP.21.02 to TT).

## Conflict of Interest

The authors declare that the research was conducted in the absence of any commercial or financial relationships that could be construed as a potential conflict of interest.

## Publisher's Note

All claims expressed in this article are solely those of the authors and do not necessarily represent those of their affiliated organizations, or those of the publisher, the editors and the reviewers. Any product that may be evaluated in this article, or claim that may be made by its manufacturer, is not guaranteed or endorsed by the publisher.
